# Supplementary material for: The Impact of Radiotherapy and Attenuated Chemotherapy Regimens in Older Patients with Classic Hodgkin Lymphoma: A Real-Life Study from the ReLLi Network
Source: Cancers (Basel). 2025 Feb 24;17(5):765. doi: 10.3390/cancers17050765 (PMC11898453; doi:10.3390/cancers17050765)
Supplement: Supplementary file 1 [file cancers-17-00765-s001.zip › cancers-3461650-supplementary.pdf]

Supplemental

The Impact of Radiotherapy and Attenuated Chemotherapy Regimens in Older Patients with Classic Hodgkin Lymphoma: A Real-Life Study from the ReLLi Network

Maria Christina Cox [1], Matteo Caridi [2,3,†], Alexandro Patirelis [4,†], Ilaria Del Giudice [5,6], Alessandro Pulsoni [5,7], Daniela Renzi [8], Sabrina Pelliccia [9], Roberta Battistini [10], Paola Anticoli Borza [11], Ombretta Annibali [12], Vito Rapisarda [1], Eleonora Alma [13], Nadia Messina [13], Gianna Maria D'Elia [5,6], Francesco Marchesi [8], Natalia Cenfra [5,7], Maria Paola Bianchi [9], Fiammetta Natalino [2], Andrea Carpaneto [10], Giovanni Manfredi Assanto [5,6], Anna Giulia Zizzari [1], Elena Maiolo [13], Vitaliana De Sanctis [14], Stefan Hohaus [13,*] and Luigi Rigacci [12]

[1] Hematology, Department of Hematology and Oncology, Tor Vergata University Hospital, 00133 Rome, Italy
[2] Division of Hematology, Belcolle Hospital, 01100 Viterbo, Italy
[3] Institute of Hematology and Center for Hemato-Oncology Research, University of Perugia and Santa Maria Della Misericordia Hospital, 06129 Perugia, Italy
[4] Thoracic Surgery, Department of Surgical Sciences, University of Rome Tor Vergata, 00133 Rome, Italy
[5] Hematology, Department of Translational and Precision Medicine, Sapienza University of Rome, 00185 Rome, Italy
[6] UOC Ematologia, AOU Policlinico Umberto I, 00161 Rome, Italy
[7] UOC Ematologia, Ospedale Santa Maria Goretti, 04100 Latina, Italy
[8] Hematology and Stem Cell Transplant Unit, IRCCS Regina Elena National Cancer Institute, 00144 Rome, Italy
[9] Hematology, Azienda Ospedaliera Universitaria Sant'Andrea, 00189 Rome, Italy
[10] Hematology, Azienda Ospedaliera San Camillo, 00152 Rome, Italy
[11] Azienda Ospedaliera San Giovanni Rome, 00184 Rome, Italy
[12] Hematology, Campus Biomedico, 00128 Rome, Italy
[13] Hematology, IRCCS Policlinico Gemelli, Università Cattolica del Sacro Cuore, 00168 Rome, Italy
[14] Radiotherapy, Azienda Ospedaliera Universitaria Sant'Andrea, 00189 Rome, Italy
* Correspondence: stefan.hohaus@unicatt.it
† These authors contributed equally to this work.

Table S1. Progression-free, overall and cancer specific survival.

		PFS (months)		OS (months)		CSS (months)	
	n	Median	5-years	Median	5-years	Median	5-years
60-69 years	68	NR	77.7%	NR	86.8%	NR	89.4%
≥70 years	61	86 (49-123)	55.7%	91 (66-116)	67.2%	105 (87-123)	69.0%

CSS: cancer specific survival; NR: not reached; OS: overall survival; PFS: progression free survival.

Table S2. Univariate and multivariate Cox regression analysis in 60-69 years old subset.

	Event Free Survival			Overall Survival			Cancer specific Survival		
	**Univariate**	**Multivariate**		**Univariate**	**Multivariate**		**Univariate**	**Multivariate**	
	p-value	HR (95% CI)	p-value	p-value	HR (95% CI)	p-value	p-value	HR (95% CI)	p-value
**Gender (M vs F)**	0.76	-	-	0.67	-	-	0.38	-	-
**Stage (I-II vs III-IV)**	**0.005**	1.9 (0.7-5.1)	0.19	**0.003**	1.6 (0.6-4.2)	0.35	**0.003**	1.6 (0.5-4.6)	0.40
**B symptoms (no vs yes)**	0.84	-	-	0.78	-	-	0.99	-	-
**Bulky disease (no vs yes)**	**0.011**	1.7(0.6-5.1)	0.29	**0.016**	1.6 (0.6-4.5)	0.36	**0.008**	1.8 (0.6-5.4)	0.26
**Hemoglobin (<10.5 vs ≥10.5) g/dl**	0.67	-	-	0.27	-	-	0.21	-	-
**WBC (<15000 vs ≥15000) μl^{-1}**	**0.006**	3.4 (1.3-8.6)	**0.010**	0.43	-	-	0.52	-	-
**Radiotherapy (no vs yes)**	**0.001**	6.0 (1.7-22.0)	**0.006**	**0.002**	5.6 (1.6-20.1)	**0.008**	**0.002**	8.1 (1.7-37.0)	**0.007**
**Complete remission (no vs yes)**	**<0.001**	0.2 (0.1-0.6)	**0.001**	**<0.001**	0.3 (0.1-0.8)	**0.009**	**<0.001**	0.3 (0.1-0.8)	**0.017**
**PET 2 (no vs yes)**	0.24	-	-	0.14	-	-	0.098	-	-

Table S3. Univariate and multivariate Cox regression analysis in ≥70 years old subset.

	Event Free Survival			Overall Survival			Cancer specific Survival		
	**Univariate**	**Multivariate**		**Univariate**	**Multivariate**		**Univariate**	**Multivariate**	
	p-value	HR (95% CI)	p-value	p-value	HR (95% CI)	p-value	p-value	HR (95% CI)	p-value
**Gender (M vs F)**	0.91	-	-	0.38	-	-	0.17	-	-
**Stage (I-II vs III-IV)**	0.47	-	-	0.63	-	-	0.33	-	-
**B symptoms (no vs yes)**	0.33	-	-	0.17	-	-	0.10	-	-
**Bulky disease (no vs yes)**	0.27	-	-	0.35	-	-	0.64	-	-
**Hemoglobin (<10.5 vs ≥10.5) g/dl**	0.45	-	-	0.60	-	-	0.40	-	-
**WBC (<15000 vs ≥15000) μl^{-1}**	0.38	-	-	0.64	-	-	0.15	-	-
**Radiotherapy (no vs yes)**	0.56	-	-	0.22	-	-	0.30	-	-
**Complete remission (no vs yes)**	**<0.001**	0.1 (0.06-0.4)	<0.001	**0.009**	0.2 (0.1-0.7)	**0.009**	**0.025**	0.2 (0.05-0.8)	**0.025**
**PET 2 (no vs yes)**	0.46	-	-	0.59	-	-	0.85	-	-

CI: confidence interval; F: female; HR: hazard ratio; M: male; PET: positron emission tomography; WBC: white blood cells.

Table S4. Report of Kaplan Meier curves median survivals.

MEDIAN SURVIVAL (months)		
**Kaplan Meier 1**	**Median**	**95% Confidence Interval**
<60 years	NR	-
60-69 years	NR	-
≥70 years	80.0	56.2-103.8
**Kaplan Meier 2**	**Median**	**95% Confidence Interval**
60-69 years	NR	-
≥70 years	86.0	44.2-127.8
**Kaplan Meier 3**	**Median**	**95% Confidence Interval**
60-69 years	NR	-
≥70 years	91.0	66.0-116.0
**Kaplan Meier 4**	**Median**	**95%Confidence Interval**
60-69 years	NR	-
≥70 years	105.0	86.7-123.2
**Kaplan Meier 5**	**Median**	**95%Confidence Interval**
60-69 years	NR	-
≥70 years	93.0	84.4-101.5
**Kaplan Meier 6**	**Median**	**95% Confidence Interval**
<60 years	NR	-
60-69 years	NR	-
≥70 years	56.0	41.5-70.5
**Kaplan Meier 7**	**Median**	**95% Confidence Interval**
<60 years	NR	-
60-69 years	NR	-
≥70 years	119.0	103.5-134.5
**Kaplan Meier 8**	**Median**	**95% Confidence Interval**
Attenuated therapy	NR	-
Curative therapy	NR	-
**Kaplan Meier 9**	**Median**	**95% Confidence Interval**
Attenuated therapy	37.0	5.1-68.9
Curative therapy	89.0	62.5-115.5
**Kaplan Meier 10**	**Median**	**95% Confidence Interval**
Not intention to BMT	45.0	19.1-70.8
Intention to BMT	NR	-
**Kaplan Meier 11**	**Median**	**95% Confidence Interval**
CHT	45.0	23.5-66.5
CHT + Auto	NR	-
**Kaplan Meier 12**	**Median**	**95% Confidence Interval**
No radiotherapy	109.0	96.8-121.2
Radiotherapy	NR	-
**Kaplan Meier 13**	**Median**	**95% Confidence Interval**
No radiotherapy	NR	-
Radiotherapy	NR	-
**Kaplan Meier 14**	**Median**	**95% Confidence Interval**
Stage I-II	NR	-
Stage III-IV	NR	-
**Kaplan Meier 15**	**Median**	**95% Confidence Interval**
Stage I-II	119.0	103.5-134.5
Stage III-IV	53.0	51.6-102.4

BMT: bone marrow transplantation; CHT: chemotherapy.

a) all patients

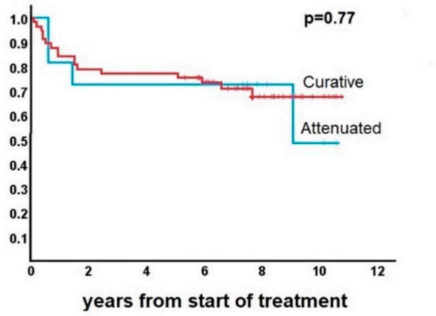

N. patients at risk of event (N. censored)

Time (years)	0	2	4	6	8	10	12
Attenuated	11(0)	8(0)	8(0)	8(0)	4(4)	2(5)	0(7)
Curative	57(0)	45(0)	44(0)	36(6)	18(22)	5(35)	0(40)

b) 60-69 years old

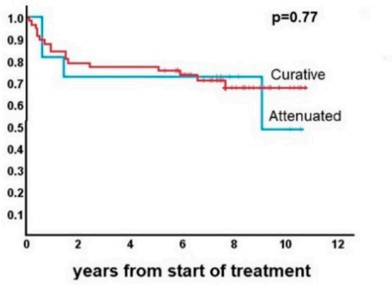

N. patients at risk of event (N. censored)

Time (years)	0	2	4	6	8	10	12
Attenuated	11(0)	8(0)	8(0)	8(0)	4(4)	2(5)	0(7)
Curative	57(0)	45(0)	44(0)	36(6)	18(22)	5(35)	0(40)

c): ≥70 years old

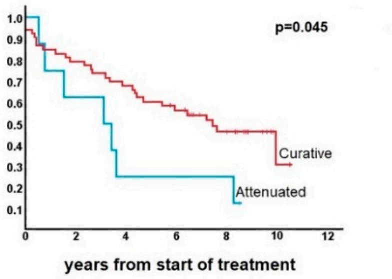

N. patients at risk of event (N. censored)

Time (years)	0	2	4	6	8	10	12
Attenuated	8(0)	5(0)	2(0)	2(0)	2(0)	0(1)	
Curative	53(0)	42(0)	36(0)	27(3)	16(10)	2(23)	0(25)

Figure S1: Event free survival in **a)** over 60 year-old, **b)** 60-69 year-old and **c)** ≥70 year-old patients who received curative or attenuated chemotherapy.

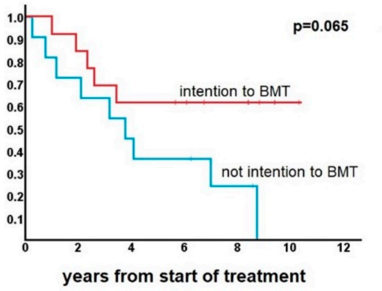

N. patients at risk of death (N. censored)

Time (years)	0	2	4	6	8	10	12
Not intention to BMT	11(0)	8(0)	5(0)	4(0)	2(1)	0(2)	
Intention to BMT	13(0)	11(0)	8(0)	7(1)	5(3)	1(7)	0(8)

BMT: bone marrow transplantation

Figure S2. Overall survival in over 60 years old patients following 2nd line treatments receiving/not marrow transplantation.
